# Assessing Efficacy of Clinical Disinfectants for Pathogenic Fungi by Single-Cell Raman Microspectroscopy

**DOI:** 10.3389/fcimb.2022.772378

**Published:** 2022-02-23

**Authors:** Fan Li, Lihui Ren, Rongze Chen, Xi Sun, Jian Xu, Pengfei Zhu, Fang Yang

**Affiliations:** ^1^ Stomatology Center, Qingdao Municipal Hospital, Qingdao, China; ^2^ School of Stomatology, Qingdao University, Qingdao, China; ^3^ Department of Pediatric Dentistry, School and Hospital of Stomatology, Tianjin Medical University, Tianjin, China; ^4^ Single-Cell Center, Chinese Academy of Science Key Laboratory of Biofuels, Shandong Key Laboratory of Energy Genetics, Shandong Energy Institute, Qingdao New Energy Shandong Laboratory, Qingdao Institute of Bioenergy and Bioprocess Technology, Chinese Academy of Sciences, Qingdao, China; ^5^ University of Chinese Academy of Sciences, Beijing, China; ^6^ College of Information Science & Engineering, Ocean University of China, Qingdao, China; ^7^ College of Biological Engineering, Tianjin Agricultural University, Tianjin, China; ^8^ Tianjin Engineering Research Center of Agricultural Products Processing, Tianjin Agricultural University, Tianjin, China

**Keywords:** heavy water, single-cell technology, *Candida albicans*, chlorhexidine gluconate, sodium hypochlorite, hydrogen peroxide

## Abstract

Disinfectants are crucial for root canal therapy (RCT), as metabolism of canal-inhabiting microbes can cause refractory infections. To develop effective yet patient- and environment-friendly disinfectant formulations, we quantitatively assessed the metabolism-inhibiting effects of intracanal disinfectants *via* D_2_O-probed Single-Cell Raman Spectra (SCRS), using *Candida albicans* (*C. albicans*) as a pathogen model. For chlorhexidine gluconate (CHX), sodium hypochlorite (NaClO), and hydrogen peroxide (H_2_O_2_), at their MIC of 4, 168, and 60 μg/ml, respectively, despite the complete growth halt, metabolic activity of individual fungal cells was reduced on average by 0.4%, 93.9%, and 94.1% at 8 h, revealing a “nongrowing but metabolically active” (NGMA) state that may underlie potential refractory infections, particularly for CHX. In contrast, at their Metabolic Activity-based Minimum Inhibitory Concentrations (MIC-MA) of 8, 336, and 120 μg/ml, respectively, metabolic activity of all cells was completely halted throughout 8 h exposure. Moreover, combined use of NaClO+H_2_O_2_ (combination at 0.5× MIC-MA each) outperforms solo uses of CHX, NaClO, H_2_O_2_, or other binary combinations. Furthermore, dynamics of SCRS revealed distinct fungicidal mechanisms of CHX, NaClO, H_2_O_2_, and their pairwise combinations. MIC-MA is advantageous in critically assessing antifungal efficacy, and NaClO+H_2_O_2_ can potentially serve as a more efficient disinfectant formula for fungal pathogens.

## Introduction

Microbial infections in the pulp and periapical tissues could cause pulpitis, apical periodontitis, or even persistent inflammatory reaction. In these endodontic infections, pathogenic fungi such as *Candida albicans* (*C. albicans*) are the most frequently isolated eukaryotes (Siqueira and Sen, 2004; Kumar et al., 2015). In particular, *C. albicans* can readily form biofilms (Alshanta et al., 2019) colonize dentinal walls, and penetrate into dentinal tubules (Siqueira et al., 2002), resulting in persistent infections (Mergoni et al., 2018). Therefore, one key goal of root canal treatment (RCT) is to control and prevent microbial infections in the intracanal areas.

During RCT, mechanical debridement by hand and rotary instruments can leave 35% or more surface area of canals untouched (Tomson and Simon, 2016). Therefore, the use of liquid intracanal disinfectants such as chlorhexidine gluconate (CHX), sodium hypochlorite (NaClO), and hydrogen peroxide (H_2_O_2_), to ensure thoroughness of the intricate debridement of intracanal and accessory canals, is a vital step of RCT and key to the final endodontic treatment success (Ahmed and Dummer, 2018). An ideal intracanal disinfectant should be effective in inhibiting the metabolism of all microbial members, capable of dissolving pulp tissue remnant and smear layer, and, moreover, nontoxic or nonallergic (Galler, 2016). However, frequently, these desirable features are mutually exclusive: e.g., the more potent pathogen-inhibitory effect can be linked to more severe side effects to host tissues (Gomes-Filho et al., 2008). Therefore, the choice of type, concentration, duration, or combination of intracanal disinfectants is often based on empirical notion, due to the inability to rapidly assess the interaction between disinfectants and microbial cells (Chong and Pitt Ford, 1992; Rahimi et al., 2014). Therefore, there is an urgent need for method development to tackle this challenge.

Current methods that assess efficacy of disinfectants and other antimicrobials can be broadly classified as “growth-based” or “non-growth-based”. Growth-based methods typically examine the sensitivity of cell growth curve to antimicrobial *via* dilution and diffusion, and quantitative parameters such as “minimum inhibitory concentration” [MIC, i.e., the lowest drug concentration under which microbial growth is entirely inhibited (Brauner et al., 2016)] are then derived. As growth inhibition does not necessarily correlate with metabolic inhibition or cell death, the growth-based methods are usually unable to distinguish between bactericidal and bacteriostatic effects and consequentially fail to detect “non-growing but metabolically active” (NGMA) cells (Tao et al., 2017), which are responsible for many latent or recurring infections (due to their ability to resume growth after removal of antimicrobials) and eventually lead to treatment failure (Manina and McKinney, 2013). Moreover, these methods can be time-consuming (frequently exceeding 24 h for fast-growing pathogens as extended duration of drug exposure to detect growth changes is required).

Rather than assessing the efficacy of antimicrobials based on “growth” inhibition, we recently introduced D_2_O-probed Single-cell Raman Microspectroscopy (D_2_O-Ramanometry), which can serve as a quantitative yet universal method to detect and measure metabolic-activity change of cells in response to drug treatments at the single-cell resolution (Teng et al., 2016; Tao et al., 2017; Bauer et al., 2020). Specifically, we proposed “Minimum Inhibitory Concentration based on Metabolic Activity” (MIC-MA), defined as the minimal dose under which the median ΔC-D-ratio at 8 h of drug exposure is ≤0 and the Standard Deviation (SD) of the ΔC-D ratio among individual cells is ≤0.005, to evaluate the metabolism-inhibiting efficacy of antimicrobials (Tao et al., 2017). However, this method and concept have not been tested in pathogenic fungi or for disinfectants; thus, it is unclear whether and how such metabolic-activity-based fungus–disinfectant interaction, including its inter-cellular heterogeneity, can be quantitatively accessed and screened for rational development of efficient disinfecting formula.

To tackle this challenge, here employing *C. albicans* as a model of fungal pathogen, we quantitatively assessed the metabolism-inhibiting effects of clinical intracanal disinfectants *via* D_2_O-probed Single-Cell Raman Spectra (SCRS), aiming to demonstrate application of the method in screening new formula of intracanal disinfectants of reliable antimicrobial efficacy.

## Results

### Tracking D_2_O Incorporation in *C. albicans via* Single-Cell Raman Spectra

**Figure 1:**
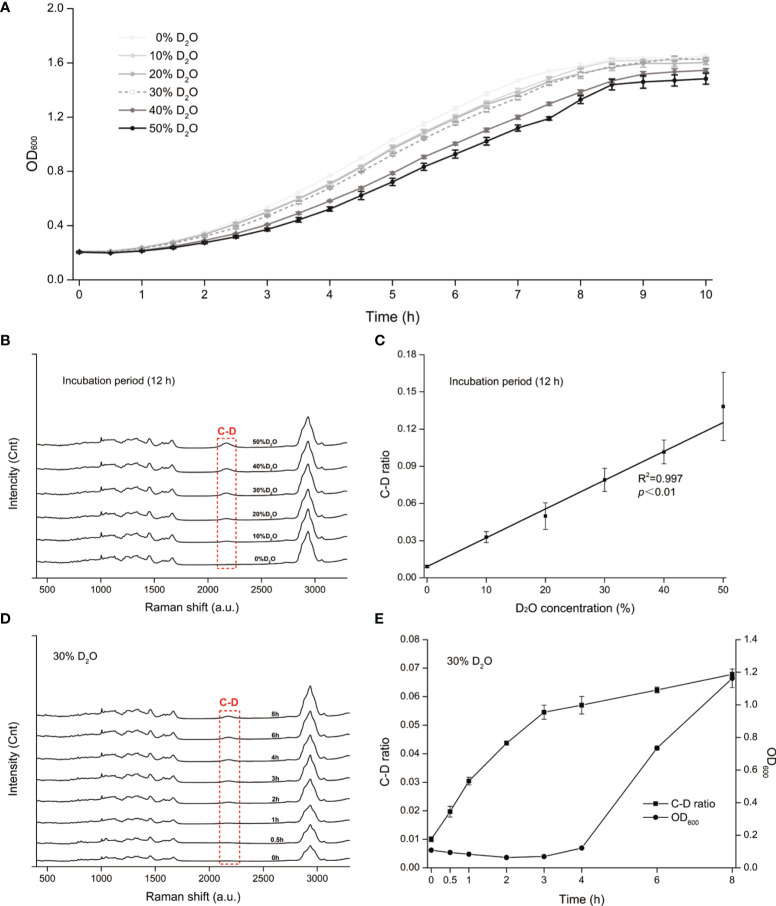
D_2_O-Labeled Single-Cell Raman Microspectroscopy of *Candida albicans* (*C. albicans*). **(A)** Temporal change of OD_600_ for the *C. albicans*, under various D_2_O doses. **(B)** Change of SCRS under increasing levels of D_2_O. *C. albicans* was grown in the medium supplemented with various levels of D_2_O overnight to reach the stationary phase, followed by SCRS acquisition. **(C)** Correlation between the C-D ratio and the D_2_O concentration for *C. albicans*. The experimental strains were grown respectively under a series of D_2_O levels and then cultured for 12 h, followed by SCRS measurement. **(D)** Temporal change of SCRS for *C. albicans* under 30% D_2_O. Graduate emergence of the C-D peak in SCRS was apparent under drug-free cultures. **(E)** Temporal change of C-D ratio and OD_600_ of *C. albicans* under 30%. The CD ratio curve is distinct from the OD_600_-based growth curve. Error bars indicate standard deviation among three biological replicates.

All living cells consume H_2_O in the metabolism process and the H_2_O intake rate is proportional to the level of cellular metabolic activity (Berry et al., 2015). Thus, metabolic inhibiting effect of an antimicrobial to a cell can be quantified based on the H_2_O intake rate of the cell under drug exposure, which is measured *via* the extent of Raman shift at the C-D (carbon-deuterium vibration) band in 2,040 to 2,300 cm^−1^ in the SCRS of a cell to which D_2_O is fed (Berry et al., 2015). However, high levels of D_2_O, which accelerate cellular D_2_O intake and thus allow faster assays, can be cytotoxic or cytostatic to cells (Takeda et al., 1998). Therefore, we started by selecting a proper concentration of D_2_O, *via* comparison among the growth curves of *C. albicans* under various D_2_O levels (**Figure 1A**). Compared with the control group (D_2_O-free conditions), *C. albicans* growth was not significantly inhibited by a D_2_O level below 30% during 10 h of culture (*p* > 0.05; **Figure 1A**). After overnight culture, which corresponds to the stationary phase of *C. albicans*, intensity of the C-D band (2,040–2,300 cm^−1^) increased along with the elevation of D_2_O level in the medium (**Figure 1B**). The corresponding C-D ratio, defined as ratio of the integrated spectral intensity of the C-D band (2,040–2,300 cm^−1^) compared to the sum of the C-D band and the predominant C-H band (2,800–3,100 cm^−1^) (Tao et al., 2017), showed strong positive correlation with media D_2_O level (*R* ^2^ = 0.997, *p* < 0.01; **Figure 1C**), consistent with the positive link between substrate level and cellular substrate intake rate (i.e., metabolic activity). Therefore, 30% D_2_O was chosen for evaluating *C. albicans*’ metabolic response to the intracanal disinfectants *via* SCRS.

Under 30% D_2_O (in the media), the C-D peak emerged and increased along with duration of D_2_O incubation (**Figure 1D**). The corresponding C-D ratio started growing almost immediately after D_2_O introduction, yet in contrast, the growth of OD_600_ was not detectable until ~4 h afterwards (**Figure 1E**). These results support detecting D_2_O incorporation *via* SCRS under these conditions and can serve as a proxy for assessing the metabolic activity of *C. albicans*, and suggest that D_2_O-probed SCRS can be more sensitive and faster than OD_600_ in detecting *C. albicans* growth, which also incurs metabolic change of individual cells.

### MIC and MIC-MA of CHX, NaClO, and H_2_O_2_ for *C. albicans*

**Figure 2:**
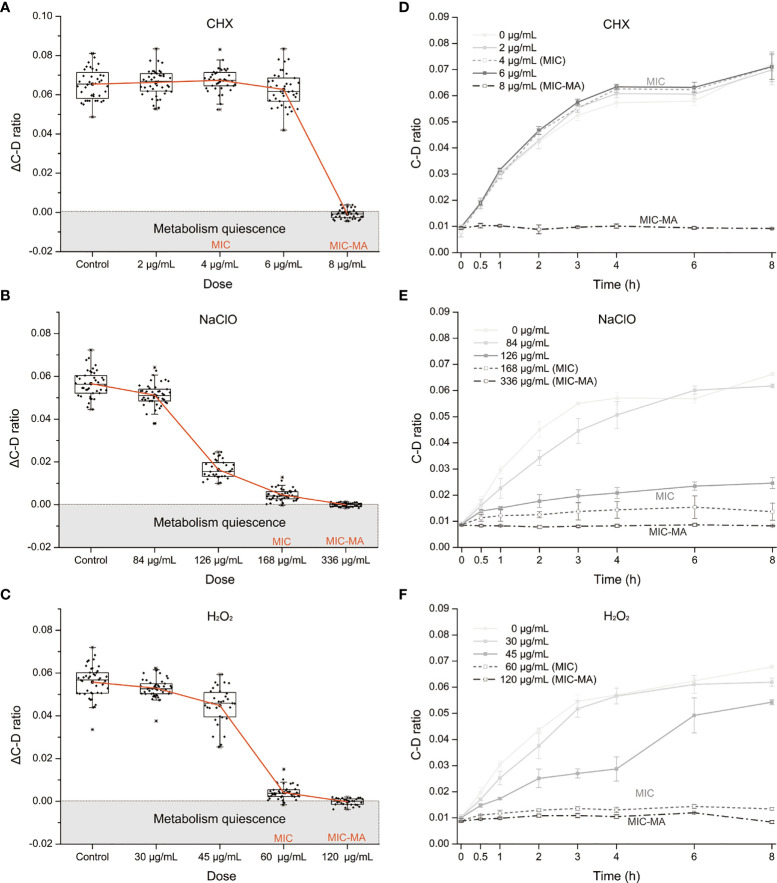
The MIC-MA for each of the three intracanal disinfectants. **(A–C)** Dose effects of CHX, NaClO, and H_2_O_2_ on the ΔC-D-ratio of *C. albicans* cells. **(D–F)** Temporal dynamics of the C-D ratio of *C. albicans* under increasing doses of CHX, NaClO, and H_2_O_2_.

To assess the metabolic susceptibility of *C. albicans* to each of the three intracanal disinfectants, we determined the corresponding MIC and MIC-MA of CHX, NaClO, and H_2_O_2_, *via* broth dilution and D_2_O-probed SCRS, respectively (**Figures 2A–F**; Materials and Methods). In contrast to MIC-MA, which assesses metabolic activity of individual cells, MIC evaluates drug efficacy based on growth inhibition of the whole bacterial population (Materials and Methods).

For CHX, the MIC and MIC-MA are 4 and 8 μg/ml, respectively (**Tables S1** and **1**). At the MIC of CHX, although fungal growth was entirely inhibited, the temporal dynamics of C-D ratio showed that it was not lower than the drug-free control and eventually reached nearly an equivalent level after 8 h treatment (*p* > 0.05; **Figure 2D**). Thus, metabolic activity of *C. albicans* cells was still quite active under the MIC of CHX (4 μg/ml; **Figures 2A, D**). For the 0.5× MIC and 1.5× MIC groups, the trend within 8 h is similar to the MIC group (*p* > 0.05; **Figure 2D**). Notably, at the MIC-MA of CHX, the C-D ratio was maintained at the baseline level within 8 h from the start of drug exposure to the end of observation period (**Figure 2D**), suggesting that the metabolism of *C. albicans* was completely inhibited (ΔC-D-ratios < 0 at 8 h; **Figure 2A**).

**Table 1:** Comparison of MIC and MIC-MA for the three antimicrobial disinfectants tested.

Disinfectant (μg/ml)	MIC	MIC-MA
CHX	4	8
NaClO	168	336
H_2_O_2_	60	120

For NaClO and H_2_O_2_, the MICs are 168 and 60 μg/ml, respectively (**Tables S1**, **1**), and its MIC-MA are 336 and 120 μg/ml, respectively, in which the averaged ΔC-D-ratios was <0 during an 8-h period (**Figures 2B, C**). The temporal dynamics of the C-D ratio showed a drug-dose-dependent effect that is reproducible (**Figures 2E, F**). At their 0.5× MICs of NaClO (84 μg/ml) and H_2_O_2_ (30 μg/ml), to a certain extent, the increment of C-D ratio was inhibited as compared to the drug-free control (*p* < 0.05). However, at their MICs of NaClO (168 μg/ml) and H_2_O_2_ (60 μg/ml), the increase of C-D ratio was much lower than the drug-free control (*p* < 0.05), and the C-D ratio was maintained at a low level but failed to reach the baseline level during the whole period. Finally, at the MIC-MAs of NaClO (336 μg/ml) and H_2_O_2_ (120 μg/ml), the C-D ratio always stayed at the baseline level (**Figures 2B, C, E, F**); thus, the metabolic activity of *C. albicans* was entirely inhibited instantaneously and throughout the 8-h duration.

### Comparison Between MIC-MAs and MICs Reveals NGMA *C. albicans* Cells

**Figure 3:**
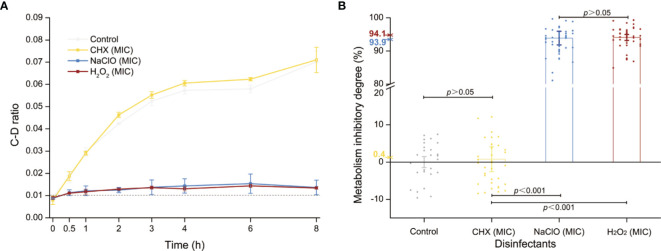
Metabolic activity of *C. albicans* under the MIC of each of the three intracanal disinfectants. **(A)** Temporal dynamics of the C-D ratio of *C. albicans* under the MIC doses. **(B)** The degree of metabolic-activity inhibition of *C. albicans* at 8 h of exposure under the MICs of CHX, NaClO, or H_2_O_2_. Each dot represents a cell.

The MICs of CHX, NaClO, and H_2_O_2_ for *C. albicans* are 4, 168, and 60 μg/ml, respectively (**Tables S1** and **1**), while the corresponding MIC-MAs are 8, 336, and 120 μg/ml (**Tables S2** and **1**). Notably, under the MIC-MA level for these three intracanal disinfectants, the majority of *C. albicans* cells have entered “metabolism quiescence zone”. However, at the MIC of CHX, C-D ratio curve was comparable to the drug-free control, showing the presence of considerable cellular metabolic activity after drug treatment. For NaClO and H_2_O_2_, under the MIC treatment, the metabolic activity of the fungal cells was inhibited to a significant degree; however, almost all fungal cells still exhibited a relatively low level of metabolic activity even after 8-h drug exposure (**Figure 3A**). Therefore, (i) the MIC-MAs of the above three intracanal disinfectants were twice that of MIC; (ii) at their respective MIC level of CHX, NaClO, and H_2_O_2_, despite the completely halted growth, metabolic activity of *C. albicans* cells was inhibited by merely 0.4%, 93.9%, and 94.1% at 8 h (i.e., the inhibitory effect of NaClO and H_2_O_2_ on the metabolic activity of *C. albicans* was much stronger than that of CHX, while no significant difference was found between NaClO and H_2_O_2_; **Figure 3B**), suggesting the presence of “nongrowing but metabolically active” (NGMA) cells that may underlie refractory infections for each of the treatments (particularly for CHX).

### Assessing the Efficacy of Disinfectant Combinations on Inhibiting Fungal Metabolism

The distinct *C. albicans*-inhibitory effects of CHX, NaClO, and H_2_O_2_ raise the possibility that rational combination of the disinfectants can potentially further improve the efficacy. To probe this hypothesis, we measured the MIC-MA of multiple combinations of different agents and concentrations.

**Figure 4:**
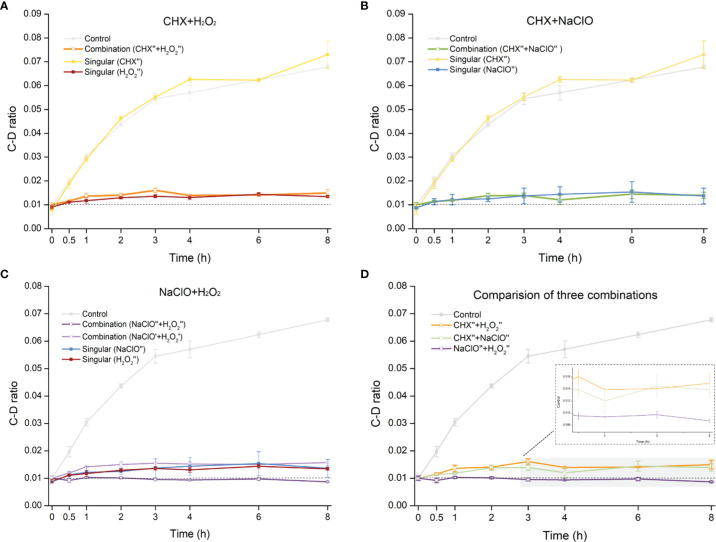
Comparison of metabolic-activity inhibition of *C. albicans* cells under various combinations of the disinfectants. Temporal dynamics of the C-D ratio of *C. albicans* under the singular disinfectant or combination of CHX and H_2_O_2_ **(A)**, CHX and NaClO **(B)**, or NaClO and H_2_O_2_ **(C)** were shown. **(D)** NaClO+H_2_O_2_ combination outperforms the combination of either CHX+H_2_O_2_ or CHX+NaClO. ´: 0.25× MIC-MA; ´´: 0.5× MIC-MA.

The CHX (0.5× MIC-MA) and H_2_O_2_ (0.5× MIC-MA) combination was unable to completely inhibit *C. albicans*’ metabolic activity, which was equivalent to the effect of using H_2_O_2_ (0.5× MIC-MA) alone (*p* > 0.05) (**Figure 4A**). The combination of CHX (0.5× MIC-MA) and NaClO (0.5× MIC-MA) was not satisfactory in inhibiting *C. albicans* metabolic activity either (**Figure 4B**). However, the metabolism of *C. albicans* can be completely inhibited by the combination of NaClO (0.5× MIC-MA) and H_2_O_2_ (0.5× MIC-MA) (**Figure 4C**). Notably, further reduction of the level of the two disinfectants, i.e., the NaClO (0.25× MIC-MA) and H_2_O_2_ (0.25× MIC-MA) combination, failed to completely inhibit the metabolic activity (**Figure 4C**). Thus, among the various singular or combinatorial recipes tested here, the combination of NaClO and H_2_O_2_ exhibits the most efficient inhibitory effect on fungal metabolism (**Figure 4D**).

### Raman Barcodes for Stress Response Provided Mechanistic Insights into Drug Efficacy

Each SCRS sampled from a drug-responding *C. albicans* population consists of thousands of Raman bands. Thus, by identifying those marker Raman bands that are both specific and shared among the six stress-response programs (Materials and Methods), we derived RBCS [Raman barcode of cellular-response to stress (Teng et al., 2016)], which consists of 48 elementary Raman bands that collectively characterize the temporal pattern of *C. albicans*’ response to each of the treatments (**Table S3**). Among them, four bands that represent carbohydrates (1,048 and 1,147 cm^−1^) and proteins (758 and 1,005 cm^−1^), respectively, were shared among the marker bands for all the six stressors; thus, they are part of a general cellular response.

**Figure 5:**
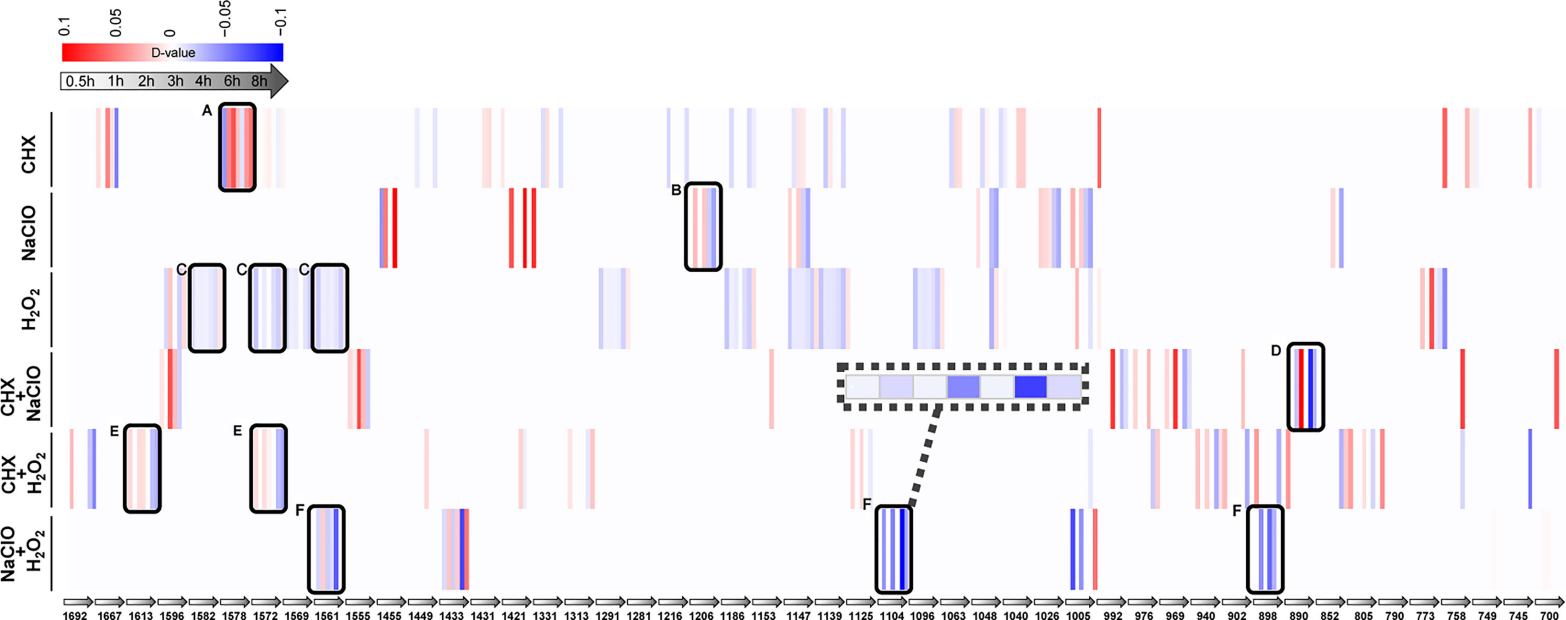
Raman-barcode of cellular-response to stressors (RBCS) under various duration of exposure to the disinfectants. The change trend of marker Raman bands under singular CHX **(A)**, NaClO **(B)**, H_2_O_2_ **(C)** or combination of CXH and NaClO **(D)**, CHX and H_2_O_2_ **(E)**, NaClO+H_2_O_2_ **(F)** were shown. The change in Raman band intensity was calculated as D-value (between stressed and control cells) and shown as blue (decreased intensity) or red (increased intensity; *p* < 0.001, Wilcoxon rank sum test).

Under CHX, the most prominent change was nucleic acid (1,578 cm^−1^) (**Figure 5A**). Compared with the control group, the intensity of nucleic acid band was generally elevated upon exposure to CHX. It is possible that the density of nucleic acids gradually decreases with the growth of cells in normal-growing cells, while CHX can alter cell membrane integrity by electrostatic binding with the negatively charged cell wall, which results in the leakage of low-molecular-weight components (Bernardi and Teixeira, 2015). These may have caused the relative increase in intensity of nucleic acid band. Under NaClO or H_2_O_2_, temporal changes of protein bands for NaClO (1,206 cm^−1^) (**Figure 5B**) and for H_2_O_2_ (1,582, 1,572, and 1,561 cm^−1^) (**Figure 5C**) were the most pronounced. Compared with the control group, the intensity of protein bands gradually reduced along with the duration of exposure. NaClO and H_2_O_2_ can produce hypochlorous acid (HClO) (Siqueira, 1997) and O_2_ (Huang and Pik, 2014) respectively, whose strong oxidizing effect can lead to destruction of proteins and other substances.

As for the combinations, the protein bands of 890 cm^−1^ (**Figure 5D**) for CHX+NaClO and of 1,613 and 1,572 cm^−1^ (**Figure 5E**) for CHX+H_2_O_2_ were reduced. It is possible that CHX precipitates proteins (Bernardi and Teixeira, 2015) and that the strong oxidizing effect of NaClO or H_2_O_2_ on protein (Siqueira, 1997; Huang and Pik, 2014) eventually leads to the decrease of protein content or density in cells. Under NaClO+H_2_O_2_, the intensity of protein (1,561 and 1,104 cm^−1^) and nucleic acid (898 cm^−1^) both decreased (**Figure 5F**), indicative of the oxidation effect that results in decreased proteins and nucleic acids (Siqueira, 1997).

## Discussion

Proper administration of intracanal disinfectants is vital for both long-term efficacy of antimicrobial RCT and reducing side effects that compromise patient experience. For example, NaClO is one of the most widely used intracanal disinfectants due to its ability to dissolving necrotic tissue and antibacterial activity (Siqueira et al., 1999). However, the proper concentration to administer under a clinical setting is controversial, as overly high drug concentrations can reduce biocompatibility and promote irritation of periodontal and periapical tissues (Tanomaru Filho et al., 2002; Gomes-Filho et al., 2008). CHX has also been suggested as an effective antimicrobial agent for RCT; however, cytotoxic effect, allergic reaction, and extrinsic tooth or tissue staining may ensue with usage (Bernardi and Teixeira, 2015). H_2_O_2_ remains a frequently used agent in RCT despite its potential for serious complications, which include air emphysema or even systemic gas embolus (Akuji and Chambers, 2017). Therefore, it is critical to develop a methodological scheme that can (*i*) quantitatively assess the antimicrobial effect based on halting of pathogen metabolic activity, instead of just growth, for avoiding later or recurring infections (Lopatkin et al., 2019), and (*ii*) rapidly screen new formula of intracanal disinfectants of reliable antimicrobial efficacy.

The susceptibility of fungal pathogens to these three intracanal disinfectants was compared *via* MIC. For *C. albicans* ATCC 64342 (also an oral isolate), Ferguson et al. reported the MICs of CHX, NaClO, and H_2_O_2_ as <0.63 μg/ml, <10 μg/ml, and 234 μg/ml, respectively (Ferguson et al., 2002). These MICs are comparable to our measurements for *C. albicans* ATCC 10231 here, with the variation likely due to the change of *C. albicans* strains and distinction in medium composition, amount of inoculum, incubation temperature, facilities, technical skills, etc. (Mouton et al., 2018). However, MIC’s reliance on growth inhibition can be time-consuming (frequently exceeding 24 h for common pathogens (Berkow et al., 2020), and also results in the inability to detect NGMA cells of *C. albicans* whose metabolic activity may cause reinfections after clinical latency (Manina and McKinney, 2013; Manina et al., 2015; Lempp et al., 2020).

Our research group originally proposed the “MIC-MA” parameter using D_2_O-Ramanometry to evaluate the metabolism-inhibiting efficacy of drugs, which tackles the drawbacks of the growth-inhibition-based methods (Tao et al., 2017). Here, we employed MIC-MA to further assess the metabolism-level inhibition of CHX, NaClO, and H_2_O_2_ on pathogenic fungi at single-cell resolution. Metabolic activity of the *C. albicans* cell population was inhibited by merely 0.4%, 93.9%, and 94.1% at their respective MIC level of CHX, NaClO, and H_2_O_2_. In contrast, the MIC-MA dose of 8 μg/ml for CHX, 336 μg/ml for NaClO, or 120 μg/ml for H_2_O_2_ can each completely halt the metabolic activity of *C. albicans*. The MIC-MAs of the above three intracanal disinfectants are approximately twice of MICs. This result is consistent with previous literature that shows that the MIC-MAs of NaF or CHX on four prevalent members of oral microbiota (*Streptococcus mutans*, *Streptococcus gordonii*, *Streptococcus sanguinis*, and *Lactobacillus fermentum*) are 2–3 times that of MICs (Tao et al., 2017). Moreover, the *C. albicans*-metabolism inhibition of CHX features a distinct threshold in the dose effect (**Figure 2A**), yet those of NaClO and H_2_O_2_ are quite different as semi-linear dose dependency (**Figures 2B, C**). In particular, at its MIC dose when cellular growth is fully arrested, for CHX, the vast majority of the disinfectant-exposed *C. albicans* cells are still alive (metabolically active). Thus, by distinguishing NGMA *C. albicans* cells and quantifying the degree of heterogeneity in metabolic phenotypes (Zhang et al., 2018; Bauer et al., 2020), MIC-MA would be an advantageous parameter to MIC in assessing disinfectant–pathogen interaction.

Based on growth inhibition, Kuruvilla et al. showed that CHX and NaClO combined in the root canal resulted in the greater reduction of microorganisms than either alone (Kuruvilla and Kamath, 1998). In addition, an antibacterial synergistic effect between CHX and H_2_O_2_ (Heling and Chandler, 1998; Steinberg et al., 1999) and those between NaClO and H_2_O_2_ were reported (Cerioni et al., 2009). Based on metabolic inhibition, we found that the combined formula of NaClO and H_2_O_2_ at 0.5× MIC-MA of each can elicit a level of metabolic inhibition of *C. albicans* that is equivalent to solo use of either NaClO or H_2_O_2_ at their respective MIC-MA level. Since a lower level of each ingredient can reduce side effect (Tanomaru Filho et al., 2002; Gomes-Filho et al., 2008), this NaClO+H_2_O_2_ formula should be more efficient. Moreover, no apparent synergistic effect in metabolic inhibition was observed when combining CHX and either NaClO or H_2_O_2_. Therefore, growth- and metabolic-based assessment can produce linked yet distinct findings.

Moreover, as a signature for the mode of action, RBCS, which is a barcode of temporal pattern of 48 elementary Raman bands, was derived for each of the pairs of fungi–drug interaction. Compared to other single-cell stress-response profiling methods such as morphological analysis (Choi et al., 2014), fluorescence imaging-based biosensing (Shintaku et al., 2014), or transcriptomics (Islam et al., 2011), RBCS can be advantageous as (i) it rapidly yields a comprehensive and landscape-like view of molecular events of stress response in a label-free, non-disruptive, and simple manner [without the need for preexisting biomarkers (Xu et al., 2017; He et al., 2021)]; (ii) it can predict global gene expression profiles, and *vice versa*, as the SCRS and transcriptomes (e.g., *via* RNA sequencing) can be connected linearly through a shared low-dimensional subspace (Germond and Ichimura, 2018; Kobayashi-Kirschvink et al., 2018).

Notably, in order to derive a clinically relevant personalized disinfectant level, drug dosages above MIC-MAs, which are much lower than the recommended clinical concentrations (2% CHX, 1%–5.25% NaClO, 3% H_2_O_2_), should be measured not just for pure culture of *C. albicans* as was here, but also for the microbiota of an infected root canal system, which likely are much more resistant to the disinfectants in the polymicrobial biofilm state (Ricucci and Siqueira, 2010) (Swimberghe et al., 2019). The heterogeneity of drug response thus can be measured *via* SCRS, the metabolite-conversion network can be profiled *via* algorithms such as Intra-Ramanome Correlation Analysis [IRCA (He et al., 2021)], and mechanism can be decoded *via* Raman-activated cell sorting [RACS (He et al., 2019); e.g., RAGE (Xu et al., 2020) and flow-mode RACS (Wang and Xin, 2020)], to establish the links between disinfectant-susceptibility phenotype and genomes or transcriptome at single-cell resolution. Nevertheless, by developing a workflow for single-cell Raman-based interaction assay for *C. albicans* and disinfectants used in RCT, this study paves the way for culture-free, rapid, mechanism-based assessment of personalized disinfecting efficacy and screening of treatment regimens for fungal infections.

## Materials and Methods

### Fungal Strain, Media, and Disinfectants

*Candida albicans* ATCC 10231 (*C. albicans*), one of the most commonly used fungal strain for drug susceptibility testing (Fidalgo et al., 2010), was obtained from China Center of Industrial Culture Collection. This strain was inoculated on Sabouraud dextrose agar plates at 37°C for 12 h. Grown colonies were picked from the plate and incubated in RPMI 1640 culture medium (pH 7.0 ± 0.1) in an aerobic incubator at 37°C. The 20% Chlorhexidine gluconate (Macklin, Shanghai, China) and 3% hydrogen peroxide (Huanbomiao, Hebei, China) and the 5.25% sodium hypochlorite (Weizhenyuan, Fujian, China) were purchased. All media were stored at 4°C.

### Sensitivity of *C. albicans* to D_2_O Concentration

*C. albicans* cells were 1:100 diluted from the stationary-phase culture and exposed to a final concentration of 0%, 10%, 20%, 30%, 40%, or 50% D_2_O. To track fungal growth under the D_2_O concentrations, the cells were cultivated in a Bioscreen C (Lab systems, Helsinki, Finland). The working volume in the Bioscreen plate was 300 μl/well and the temperature was controlled at 37°C and optical density (OD) was controlled at 600 nm. OD of the samples was automatically read at regular intervals of 30 min, over a 10-h period (before every measurement, the sample was gently shaken for 10 s). In addition, the cells were sampled at various time points for acquisition of SCRS to probe the intake of D_2_O by *C. albicans* cells. Three biological replicates were carried out.

### Measuring the MICs of Each of the Three Intracanal Disinfectants for *C. albicans*

Several colonies from a 24-h-old *C. albicans* culture grown on Sabouraud dextrose agar plate were transferred to 5 ml of sterile water and vortexed for 15 s until evenly distributed. The measurement of MIC was performed using the broth dilution method according to Clinical Laboratory Standards Institute (CLSI) guidelines. By adjusting cell density, cell suspensions were prepared to reach 0.5 McFarland standard (1× 10^6^ to 5× 10^6^ cells/ml). Then, a working suspension was prepared *via* a 1:100 dilution followed by a 1:20 dilution of the stock suspension with RPMI 1640 to obtain a final cell density of 5× 10^2^ to 2.5× 10^3^ cells/ml. Then, the MIC value was determined by measuring the change of OD_600_ before and after a 24-h disinfectant exposure. Three biological parallels were carried out.

### Measuring the MIC-MAs for Each of the Three Intracanal Disinfectants for *C. albicans*

*C. albicans* cells were incubated at 37°C in media with the various formulas of intracanal disinfectants and 30% D_2_O. Samples were collected at 0, 0.5, 1, 2, 3, 4, 6, and 8 h after exposure, respectively, for acquisition of SCRS. The concentrations of disinfectants were initially set as 0, 1/2× MIC, MIC, and 2× MIC. Then, the change of C-D ratio of ~30 individual *C. albicans* cells randomly sampled from the population was profiled before and after drug treatment for 8 h. The approximate MIC-MA value was found when the mean value of C-D ratio at 8 h minus 0 h (ΔC-D ratio) of drug exposure is ≤0 and the SD is ≤0.005.

### Calculation of the Metabolism-Inhibiting Degree of the Disinfectants (%)

The C-D ratio values of the control group (i.e., the absence of disinfectants) were employed as a reference to calculate the percentage decrease of the C-D ratio values after exposure to the intracanal disinfectants, so as to quantify the degree of metabolic inhibition. The following formula was used: 1 − x/x_0_, where x_0_ is the C-D ratio of the control group at 8 h, while x is the C-D ratio of the test group 8 h after exposure to a particular formula of the intracanal disinfectants.

### Assessment of the Efficacy of Combinations of Intracanal Disinfectants

The combination of disinfectants tested includes MIC and 0.5× MIC of CHX and H_2_O_2_, MIC and 0.5× MIC of CHX and NaClO, and MIC and 0.5× MIC of NaClO and H_2_O_2_, respectively. The negative control did not include any disinfectants. Samples were collected at 0, 0.5, 1, 2, 3, 4, 6, and 8 h after exposure respectively for SCRS acquisition. Then, the effect of *C. albicans* metabolism inhibiting was determined by measuring the change of C-D ratio value. Three biological replicates were carried out for each condition.

### Raman Microspectroscopy Analysis

Samples pretreatment and SCRS acquisition were performed as we previously described with slight modification (Teng et al., 2016; Tao et al., 2017). In brief, SCRS were obtained using a Clinical Antimicrobial Susceptibility Test Ramanometry instrument (CAST-R; Qingdao Single-Cell Biotech Inc, China) or a modified confocal Raman-fluorescent microscope based on LabRam HR system (Horiba Ltd., U.K.). The acquisition time for each cell was 1 s.

Pre-processing of raw SCRS data was performed using R (version 3.5.1), including background subtraction and area normalization. Spectra were cropped to a spectral region of interest ranging from 600 cm^−1^ to 1800 cm^−1^ for chemometrics analysis.

### Raman Barcodes for Stress Response and Chemometrics Analyses

After a series of basic processing of the raw Raman spectra (600 cm^−1^ to 1800 cm^−1^), in order to get the marker bands, the Random Forest model was firstly used to classify SCRS under different disinfectant treatments *via* default parameters [R package “randomForest”, ntree = 5,000, using default mtry of sqrt(p) where p is the number of Raman bands]. The rank lists of Raman bands in the order of “band importance” by Random Forests were determined over 50 iterations of the algorithm. Then, the SCRS datasets were reordered based on the rank list and used as the input data for calculating the minimum number (Nmin) of Raman bands for discriminating between the control and the stressed cells *via* ROC (receiver operating characteristic) analysis, based on the largest AUC (area under the ROC curve). Finally, Top Nmin ranking bands that show significant difference between the control and the disinfectant-exposed cells were designated as the marker bands for each of the disinfectant treatments (Wilcoxon rank sum test; *p* < 0.01).

## Data Availability Statement

All the Single-cell Raman Spectra produced in this study are publicly accessible at: http://pub.single-cell.cn/index.php/Publication/view_anti/P_SCRS0101.

## Author Contributions

FL, LR, PZ, and FY conceived and designed the research. FL carried out the experiments with the supervision of LR, RC, XS, JX, PZ, and FY. FL, LR, RC, XS, and PZ analyzed data. FL drew the figures. FL and LR drafted the manuscript. FL, XS, JX, PZ, and FY critically revised the manuscript. All authors contributed to the article and approved the submitted version.

## Funding

This work was supported by grants 31300424, 81670979, 31827801, 32030003, and 31701569 from the National Natural Science Foundation of China; XDB29050400 and KFJ-STS-QYZX-087 from CAS; and 2021JJ013 from Key Laboratory of Wuliangye-flavor Liquor Solid-state Fermentation, China National Light Industry. The funders had no role in the study design, data collection and analysis, decision to publish, or preparation of the manuscript.

## Conflict of Interest

The authors declare that the research was conducted in the absence of any commercial or financial relationships that could be construed as a potential conflict of interest.

## Publisher’s Note

All claims expressed in this article are solely those of the authors and do not necessarily represent those of their affiliated organizations, or those of the publisher, the editors and the reviewers. Any product that may be evaluated in this article, or claim that may be made by its manufacturer, is not guaranteed or endorsed by the publisher.

## References

[B1] Ahmed H. M. A. Dummer P. M. H. (2018). A New System for Classifying Tooth, Root and Canal Anomalies. Int. Endod. J. 51 (4), 389–404. doi: 10.1111/iej.12867 29023779

[B2] Akuji M. A. Chambers D. J. (2017). Hydrogen Peroxide: More Harm Than Good? Br. J. Anaesth 118 (6), 958–959. doi: 10.1093/bja/aex151 28575345

[B3] Alshanta O. A. Shaban S. Nile C. J. (2019). Candida Albicans Biofilm Heterogeneity and Tolerance of Clinical Isolates: Implications for Secondary Endodontic Infections. Antibiotics (Basel) 8 (4), 204. doi: 10.3390/antibiotics8040204 PMC6963865 31671533

[B4] Bauer D. Wieland K. Qiu L. Neumann-Cip A. C. Magistro G. Stief C.. (2020). Heteroresistant Bacteria Detected by an Extended Raman-Based Antibiotic Susceptibility Test. Anal Chem. 92 (13), 8722–8731. doi: 10.1021/acs.analchem.9b05387 32285664

[B5] Berkow E. L. Lockhart S. R. Ostrosky-Zeichner L. (2020). Antifungal Susceptibility Testing: Current Approaches. Clin. Microbiol. Rev. 33 (3), 00069–00019. doi: 10.1128/cmr.00069-19 PMC7194854 32349998

[B6] Bernardi A. Teixeira C. S. (2015). The Properties of Chlorhexidine and Undesired Effects of its Use in Endodontics. Quintessence Int. 46 (7), 575–582. doi: 10.3290/j.qi.a33934 25918757

[B7] Berry D. Mader E. Lee T. K. Woebken D. Wang Y. Zhu D.. (2015). Tracking Heavy Water (D2O) Incorporation for Identifying and Sorting Active Microbial Cells. Proc. Natl. Acad. Sci. U. S. A. 112 (2), E194–E203. doi: 10.1073/pnas.1420406112 25550518 PMC4299247

[B8] Brauner A. Fridman O. Gefen O. Balaban N. Q. (2016). Distinguishing Between Resistance, Tolerance and Persistence to Antibiotic Treatment. Nat. Rev. Microbiol. 14 (5), 320–330. doi: 10.1038/nrmicro.2016.34 27080241

[B9] Cerioni L. Rapisarda V. A. Hilal M. Prado F. E. Rodríguez-Montelongo L. (2009). Synergistic Antifungal Activity of Sodium Hypochlorite, Hydrogen Peroxide, and Cupric Sulfate Against Penicillium Digitatum. J. Food Prot 72 (8), 1660–1665. doi: 10.4315/0362-028x-72.8.1660 19722397

[B10] Choi J. Yoo J. Lee M. Kim E. G. Lee J. S. Lee S.. (2014). A Rapid Antimicrobial Susceptibility Test Based on Single-Cell Morphological Analysis. Sci. Transl. Med. 6 (267), 267ra174. doi: 10.1126/scitranslmed.3009650 25520395

[B11] Chong B. S. Pitt Ford T. R. (1992). The Role of Intracanal Medication in Root Canal Treatment. Int. Endod. J. 25 (2), 97–106. doi: 10.1111/j.1365-2591.1992.tb00743.x 1399059

[B12] Ferguson J. W. Hatton J. F. Gillespie M. J. (2002). Effectiveness of Intracanal Irrigants and Medications Against the Yeast Candida Albicans. J. Endod. 28 (2), 68–71. doi: 10.1097/00004770-200202000-00004 11833690

[B13] Fidalgo T. K. Barcelos R. Portela M. B. Soares R. M. Gleiser R. Silva-Filho F. C. (2010). Inhibitory Activity of Root Canal Irrigants Against Candida Albicans, Enterococcus Faecalis and Staphylococcus Aureus. Braz. Oral. Res. 24 (4), 406–412. doi: 10.1590/s1806-83242010000400006 21180960

[B14] Galler K. M. (2016). Clinical Procedures for Revitalization: Current Knowledge and Considerations. Int. Endod. J. 49 (10), 926–936. doi: 10.1111/iej.12606 26715631

[B15] Germond A. Ichimura T. (2018). Raman Spectral Signature Reflects Transcriptomic Features of Antibiotic Resistance in Escherichia Coli. Commun. Biol. 1, 85. doi: 10.1038/s42003-018-0093-8 30271966 PMC6123714

[B16] Gomes-Filho J. E. Aurelio K. G. Costa M. M. Bernabe P. F. (2008). Comparison of the Biocompatibility of Different Root Canal Irrigants. J. Appl. Oral. Sci. 16 (2), 137–144. doi: 10.1590/s1678-77572008000200011 19089206 PMC4327634

[B17] He Y. Huang S. Zhang P. Ji Y. Xu J. (2021). Intra-Ramanome Correlation Analysis Unveils Metabolite Conversion Network From an Isogenic Population of Cells. mBio 12 (4), e0147021. doi: 10.1128/mBio.01470-21 34465024 PMC8406334

[B18] Heling I. Chandler N. P. (1998). Antimicrobial Effect of Irrigant Combinations Within Dentinal Tubules. Int. Endod. J. 31 (1), 8–14. doi: 10.1046/j.1365-2591.1998.t01-1-00124.x 9823123

[B19] He Y. Wang X. Ma B. Xu J. (2019). Ramanome Technology Platform for Label-Free Screening and Sorting of Microbial Cell Factories at Single-Cell Resolution. Biotechnol. Adv. 37 (6), 107388. doi: 10.1016/j.biotechadv.2019.04.010 31152870

[B20] Huang C. Pik J. (2014). Tension Pneumocephalus and Oxygen Emboli From Hydrogen Peroxide Irrigation. J. Clin. Neurosci. 21 (2), 323–325. doi: 10.1016/j.jocn.2012.10.044 23751899

[B21] Islam S. Kjällquist U. Moliner A. Zajac P. Fan J. B. Lönnerberg P.. (2011). Characterization of the Single-Cell Transcriptional Landscape by Highly Multiplex RNA-Seq. Genome Res. 21 (7), 1160–1167. doi: 10.1101/gr.110882.110 21543516 PMC3129258

[B22] Kobayashi-Kirschvink K. J. Nakaoka H. Oda A. Kamei K. F. Nosho K. Fukushima H.. (2018). Linear Regression Links Transcriptomic Data and Cellular Raman Spectra. Cell Syst. 7 (1), 104–117.e104. doi: 10.1016/j.cels.2018.05.015 29936183

[B23] Kumar J. Sharma R. Sharma M. Prabhavathi V. Paul J. Chowdary C. D. (2015). Presence of Candida Albicans in Root Canals of Teeth With Apical Periodontitis and Evaluation of Their Possible Role in Failure of Endodontic Treatment. J. Int. Oral. Health 7 (2), 42–45. PMC4377149 25859106

[B24] Kuruvilla J. R. Kamath M. P. (1998). Antimicrobial Activity of 2.5% Sodium Hypochlorite and 0.2% Chlorhexidine Gluconate Separately and Combined, as Endodontic Irrigants. J. Endod. 24 (7), 472–476. doi: 10.1016/S0099-2399(98)80049-6 9693573

[B25] Lempp M. Lubrano P. Bange G. Link H. (2020). Metabolism of non-Growing Bacteria. Biol. Chem. 401 (12), 1479–1485. doi: 10.1515/hsz-2020-0201 32845858

[B26] Lopatkin A. J. Stokes J. M. Zheng E. J. Yang J. H. (2019). Bacterial Metabolic State More Accurately Predicts Antibiotic Lethality Than Growth Rate. Nat. Microbiol. 4 (12), 2109–2117. doi: 10.1038/s41564-019-0536-0 31451773 PMC6879803

[B27] Manina G. Dhar N. McKinney J. D. (2015). Stress and Host Immunity Amplify Mycobacterium Tuberculosis Phenotypic Heterogeneity and Induce Nongrowing Metabolically Active Forms. Cell Host Microbe 17 (1), 32–46. doi: 10.1016/j.chom.2014.11.016 25543231

[B28] Manina G. McKinney J. D. (2013). A Single-Cell Perspective on non-Growing But Metabolically Active (NGMA) Bacteria. Curr. Top. Microbiol. Immunol. 374, 135–161. doi: 10.1007/82_2013_333 23793585

[B29] Mergoni G. Percudani D. Lodi G. Bertani P. Manfredi M. (2018). Prevalence of Candida Species in Endodontic Infections: Systematic Review and Meta-Analysis. J. Endod. 44 (11), 1616–1625.e1619. doi: 10.1016/j.joen.2018.07.016 30241680

[B30] Mouton J. W. Muller A. E. Canton R. Giske C. G. Kahlmeter G. Turnidge J. (2018). MIC-Based Dose Adjustment: Facts and Fables. J. Antimicrob. Chemother. 73 (3), 564–568. doi: 10.1093/jac/dkx427 29216348

[B31] Rahimi S. Janani M. Lotfi M. Shahi S. Aghbali A. Vahid Pakdel M.. (2014). A Review of Antibacterial Agents in Endodontic Treatment. Iran Endod. J. 9 (3), 161–168. 25031587 PMC4099945

[B32] Ricucci D. Siqueira J. F. Jr. (2010). Biofilms and Apical Periodontitis: Study of Prevalence and Association With Clinical and Histopathologic Findings. J. Endod. 36 (8), 1277–1288. doi: 10.1016/j.joen.2010.04.007 20647081

[B33] Shintaku H. Nishikii H. Marshall L. A. Kotera H. Santiago J. G. (2014). On-Chip Separation and Analysis of RNA and DNA From Single Cells. Anal. Chem. 86 (4), 1953–1957. doi: 10.1021/ac4040218 24499009

[B34] Siqueira J. J. (1997). Evaluation of the Effectiveness of Sodium Hypochlorite Used With Three Irrigation Methods in the Elimination of Enterococcus Faecalis From the Root Canal, *In Vitro* . Int. Endod. J. 30 (4), 279–282. doi: 10.1046/j.1365-2591.1997.00096.x 9477814

[B35] Siqueira J. F. Lima K. C. Magalhães F. A. C. Lopes H. P. Uzeda M. D. (1999). Mechanical Reduction of the Bacterial Population in the Root Canal by Three Instrumentation Techniques. J. Endod. 25 (5), 332–335. doi: 10.1016/S0099-2399(06)81166-0 10530256

[B36] Siqueira J. F. Jr. Rôças I. N. Lopes H. P. Elias C. N. de Uzeda M. (2002). Fungal Infection of the Radicular Dentin. J. Endod. 28 (11), 770–773. doi: 10.1097/00004770-200211000-00006 12470022

[B37] Siqueira J. F. Jr. Sen B. H. (2004). Fungi in Endodontic Infections. Oral. Surg. Oral. Med. Oral. Pathol. Oral. Radiol. Endod. 97 (5), 632–641. doi: 10.1016/s1079210404000046 15153878

[B38] Steinberg D. Heling I. Daniel I. Ginsburg I. (1999). Antibacterial Synergistic Effect of Chlorhexidine and Hydrogen Peroxide Against Streptococcus Sobrinus, Streptococcus Faecalis and Staphylococcus Aureus. J. Oral. Rehabil. 26 (2), 151–156. doi: 10.1046/j.1365-2842.1999.00343.x 10080313

[B39] Swimberghe R. C. D. Coenye T. De Moor R. J. G. (2019). Biofilm Model Systems for Root Canal Disinfection: A Literature Review. Int. Endod. J. 52 (5), 604–628. doi: 10.1111/iej.13050 30488449

[B40] Takeda H. Nio Y. Omori H. Uegaki K. Hirahara N. Sasaki S.. (1998). Mechanisms of Cytotoxic Effects of Heavy Water (Deuterium Oxide: D2O) on Cancer Cells. Anticancer Drugs 9 (8), 715–725. doi: 10.1097/00001813-199809000-00007 9823430

[B41] Tanomaru Filho M. Leonardo M. R. Silva L. A. Anibal F. F. Faccioli L. H. (2002). Inflammatory Response to Different Endodontic Irrigating Solutions. Int. Endod. J. 35 (9), 735–739. doi: 10.1046/j.1365-2591.2002.00544.x 12449023

[B42] Tao Y. Wang Y. Huang S. Zhu P. Huang W. E. Ling J.. (2017). Metabolic-Activity-Based Assessment of Antimicrobial Effects by D2O-Labeled Single-Cell Raman Microspectroscopy. Anal. Chem. 89 (7), 4108–4115. doi: 10.1021/acs.analchem.6b05051 28282113

[B43] Teng L. Wang X. Wang X. Gou H. Ren L. Wang T.. (2016). Label-Free, Rapid and Quantitative Phenotyping of Stress Response in E. Coli *via* Ramanome. Sci. Rep. 6, 34359. doi: 10.1038/srep34359 27756907 PMC5069462

[B44] Tomson P. L. Simon S. R. (2016). Contemporary Cleaning and Shaping of the Root Canal System. Prim Dent. J. 5 (2), 46–53. doi: 10.1308/205016816819304196 28826433

[B45] Wang X. Xin Y. (2020). Positive Dielectrophoresis-Based Raman-Activated Droplet Sorting for Culture-Free and Label-Free Screening of Enzyme Function *In Vivo* . Sci. Adv. 6 (32), eabb3521. doi: 10.1126/sciadv.abb3521 32821836 PMC7413728

[B46] Xu T. Gong Y. Su X. Zhu P. Dai J. Xu J.. (2020). Phenome-Genome Profiling of Single Bacterial Cell by Raman-Activated Gravity-Driven Encapsulation and Sequencing. Small 16 (30), e2001172. doi: 10.1002/smll.202001172 32519499

[B47] Xu J. Ma B. Su X. Huang S. Xu X. Zhou X.. (2017). Emerging Trends for Microbiome Analysis: From Single-Cell Functional Imaging to Microbiome Big Data. Engineering 3 (1), 66–70. doi: 10.1016/j.eng.2017.01.020

[B48] Zhang S. Guo L. Yang K. Zhang Y. Ye C. Chen S.. (2018). Induction of Escherichia Coli Into a VBNC State by Continuous-Flow UVC and Subsequent Changes in Metabolic Activity at the Single-Cell Level. Front. Microbiol. 9. doi: 10.3389/fmicb.2018.02243 PMC6167417 30319570

