# Supplementary material for: Assessing Efficacy of Clinical Disinfectants for Pathogenic Fungi by Single-Cell Raman Microspectroscopy
Source: Front Cell Infect Microbiol. 2022 Feb 23;12:772378. doi: 10.3389/fcimb.2022.772378 (PMC8905662; doi:10.3389/fcimb.2022.772378)
Supplement: Supplementary file 1 [file DataSheet_1.docx]

Supplementary Material

**Table S1. MIC of three disinfectants for the clinical fungal strain of** ***Candida albicans* ATCC 10231 (*C.albicans*).** Note: MIC, highlighted as gray, was the lowest drug dose under which ΔOD_600_ was < 0.05.

| *C.albicans* | **CHX (μg/mL)** | | | | | | |
| --- | --- | --- | --- | --- | --- | --- | --- |
|  | **0** | **1** | **2** | **4** | **8** | **16** | **32** |
| **ΔOD_600_** | **0.1487** | **0.137** | **0.115** | **0.001** | **0.0003** | **0.0003** | **0.001** |
| *C.albicans* | **NaClO (μg/mL )** | | | | | | |
|  | **0** | **42** | **84** | **168** | **336** | **672** | **1334** |
| **ΔOD_600_** | **0.1487** | **0.112** | **0.103** | **0.004** | **0.001** | **0.0017** | **0.003** |
| *C.albicans* | **H_2_O_2_ (μg/mL )** | | | | | | |
|  | **0** | **30** | **60** | **120** | **240** | **480** | **960** |
| **ΔOD_600_** | **0.1487** | **0.135** | **0.043** | **0** | **0.001** | **0.002** | **0.003** |

**Table S2. MIC-MA of the three disinfectants for the clinical fungal strain of *C.albicans*.** Note: MIC-MA, highlighted as gray, was the lowest drug concentration under which ΔC-D ratio was ≤ 0 and SD ≤ 0.005. ΔC-D ratio represents the change of the C-D ratio after 8 hours of disinfectants treatment.

| *C.albicans* | **CHX (μg/mL)** | | | | |
| --- | --- | --- | --- | --- | --- |
|  | **0** | **2** | **4** | **6** | **8** |
| **ΔC-D ratio** | **0.065411** | **0.066321** | **0.071364** | **0.062691** | **-0.00089** |
| *C.albicans* | **NaClO (μg/mL)** | | | | |
|  | **0** | **84** | **126** | **168** | **336** |
| **ΔC-D ratio** | **0.056562** | **0.051214** | **0.016402** | **0.004585** | **-3.5E-05** |
| *C.albicans* | **H_2_O_2_ (μg/mL)** | | | | |
|  | **0** | **30** | **45** | **60** | **120** |
| **ΔC-D ratio** | **0.055728** | **0.052872** | **0.044774** | **0.004117** | **-0.00023** |

**Table S3. The 48 Raman bands that underlie RBCS for the response of *C.albicans* cells to the three disinfectants.** Raman bands that changed significantly were labeled as “●”, while those shared among the three disinfectants were underlined.

| Raman bands  (cm^-1^) | Assignments | Role in stress response | | | | | | | | |
| --- | --- | --- | --- | --- | --- | --- | --- | --- | --- | --- |
|  |  | CHX | H_2_O_2_ | NaClO | CHX+  H_2_O_2_ | | | CHX+  NaClO | | NaClO+  H_2_O_2_ |
| ~1692 | proteins;Amide I (turns and bands) |  |  |  | | ● |  | | ● | |
| ~1667 | proteins;Protein band;C-C stretching band;д┴-Helical structure of amide I;Structural protein modes of tumors;Carbonyl stretch (C-O) | ● |  | ● | |  |  | |  | |
| ~1613 | proteins;Tyrosine | ● |  |  | | ● |  | |  | |
| ~1596 | others;C=N and C-C stretching in quinoid ring |  | ● |  | |  | ● | |  | |
| ~1582 | proteins;д─(C-C), phenylalanine;Phenylalanine |  | ● |  | |  | ● | |  | |
| ~1578 | nucleic_acids;Guanine (N3);Guanine, adenine | ● | ● | ● | |  |  | |  | |
| ~1572 | nucleic_acids;proteins;Guanine, adenine, TRP (protein) | ● | ● |  | | ● |  | |  | |
| ~1569 | Unknown |  | ● |  | |  |  | |  | |
| ~1561 | proteins;Tryptophan |  | ● |  | | ● | ● | | ● | |
| ~1555 | proteins;Amide II | ● |  |  | |  | ● | |  | |
| ~1449 | proteins;lipids;C-H vibration (proteins);C-H vibration (lipids);Lipids | ● |  |  | | ● |  | |  | |
| ~1455 | nucleic_acids;Deoxyribose;д─(CH2) |  |  | ● | |  |  | |  | |
| ~1433 | others;CH2 scissoring |  |  |  | | ● |  | | ● | |
| ~1431 | Unknown | ● |  | ● | |  |  | |  | |
| ~1421 | nucleic_acids;A, G (ring breathing modes of the DNA/RNA bases) |  |  | ● | | ● |  | |  | |
| ~1331 | others;C-C stretch of phenyl (1) and C3-C3 stretch and C5-C5 stretch CHд┴ in-plane bend | ● |  |  | |  | ● | |  | |
| ~1313 | lipids;proteins;CH3CH2 twisting mode of collagen/lipid |  | ● |  | | ● |  | |  | |
| ~1291 | Unknown |  | ● |  | |  |  | |  | |
| ~1281 | Unknown;Unknown |  |  |  | |  |  | | ● | |
| ~1216 | others;Stretching of CN | ● |  | ● | |  |  | |  | |
| ~1206 | proteins;Hydroxyproline, tyrosine (collagen assignment) |  |  | ● | |  |  | |  | |
| ~1186 | Unknown | ● | ● |  | |  |  | |  | |
| ~1153 | carbohydrates;Carbohydrates peak for solutions |  |  | ● | |  | ● | |  | |
| ~1147 | carbohydrates;Carbohydrates peak for solids | ● | ● | ● | | ● | ● | | ● | |
| ~1139 | Unknown | ● | ● |  | |  |  | |  | |
| ~1125 | carbohydrates;C-O-H deformation,C-O and C-C stretches |  | ● |  | | ● |  | |  | |
| ~1104 | proteins;Phenylalanine (proteins) |  |  |  | |  |  | | ● | |
| ~1096 | nucleic_acids;Phosphodioxy (PO2-) groups | ● | ● | ● | |  | ● | |  | |
| ~1063 | lipids;C-C skeletal stretch random conformation | ● |  |  | |  |  | |  | |
| ~1048 | carbohydrates;Glycogen | ● | ● | ● | | ● | ● | | ● | |
| ~1040 | Unknown | ● |  |  | |  |  | |  | |
| ~1026 | carbohydrates;Carbohydrates peak for solutions;Glycogen |  |  | ● | |  |  | |  | |
| ~1005 | proteins;carotenoids;Phenylalanine ring breath,carotene C-H bending | ● | ● | ● | | ● | ● | | ● | |
| ~992 | proteins;Single human RBC, phenylalanine, NADH |  |  |  | |  | ● | |  | |
| ~976 | nucleic_acids;Ribose vibration, one of the distinct RNA modes (with 874 and 915 cm-1) |  |  |  | | ● | ● | | ● | |
| ~969 | proteins;nucleic_acids;Phosphate monoester groups of phosphorylated proteins and cellular nucleic acids |  |  |  | |  | ● | |  | |
| ~940 | carbohydrates;C-O-C and C-O-H deformations; C-O stretching |  |  |  | | ● |  | |  | |
| ~902 | others;C-C skeletal stretching |  |  |  | | ● |  | | ● | |
| ~898 | carbohydrates;nucleic_acids;Monosaccharides (д┬-glucose), (C-O-C) skeletal mode;Disaccharide (maltose), (C-O-C) skeletal mode;Adenine |  |  |  | | ● | ● | | ● | |
| ~890 | proteins;Protein bands;Structural protein modes of tumors;д┬-Anomers |  | ● |  | |  | ● | |  | |
| ~852 | proteins;carbohydrates;Proline, hydroxyproline, tyrosine;Tyrosine ring breathing;Glycogen |  |  | ● | | ● | ● | |  | |
| ~805 | nucleic_acids;Uracil-based ring breathing mode |  |  |  | | ● | ● | | ● | |
| ~790 | nucleic_acids;Pyrimidine |  | ● |  | |  |  | |  | |
| ~773 | lipids;Phosphatidylinositol | ● | ● |  | |  |  | |  | |
| ~758 | proteins;Tryptophan;Ethanolamine group;Phosphatidylethanolamine | ● | ● | ● | | ● | ● | | ● | |
| ~749 | proteins;Symmetric breathing of tryptophan (protein assignment) |  | ● |  | | ● | ● | | ● | |
| ~745 | nucleic_acids;T (ring breathing mode of DNA/RNA bases) | ● |  |  | | ● |  | | ● | |
| ~700 | lipids;Cholesterol, cholesterol ester |  |  |  | | ● | ● | | ● | |
